# Supplementary material for: Relative Frequencies of PAX6 Mutational Events in a Russian Cohort of Aniridia Patients in Comparison with the World’s Population and the Human Genome
Source: Int J Mol Sci. 2022 Jun 15;23(12):6690. doi: 10.3390/ijms23126690 (PMC9223373; doi:10.3390/ijms23126690)
Supplement: Supplementary file 1 [file ijms-23-06690-s001.zip › Table S1.pdf]

Table S1. Summary of 31 newly reported *PAX6* intragenic mutations.

Novel/ Previously reported	Nucleotide change	Exon	Predicted effect on protein	PAX6 domain	Proband / Familial or *de novo* case	ACMG variant status classification (evidence criteria)
novel	c.19_31del	Ex 5	p.(Gly7Serfs*20)	PD	T-117.03/*de novo*	Very strong evidence for pathogenic (PVS1)
Previously reported PubMed 27081561	c.109dup	Ex 5	p.(Ala37Glyfs*19)	PD	89.03/*de novo*	Very strong evidence for pathogenic (PVS1)
Previously reported PubMed:27081561	c.141+2T>G	Int 6	r.spl	–	AN215/*de novo*	Very strong evidence for pathogenic (PVS1)
Previously reported PubMed: 7958875	c.142-139T>C	Int 5	r.spl	–	AN123/*de novo*	Very strong evidence for pathogenic (PVS1)

Previously reported PubMed: 34065151	c.177G>T	Ex 6	p.(Arg59Ser)	PD	032.1/fam(mat)	Pathogenic (PM2, PS1, PM1, PP1, PP5, PP4)
Previously reported PubMed: 29217025	c.270T>A	Ex 6	p.(Tyr90*)	PD	T-179.03/*de novo*	Very strong evidence for pathogenic (PVS1)
novel	c.271A>T	Ex 6	p.(Lys91*)	PD	AN196/*de novo*	Very strong evidence for pathogenic (PVS1)
novel	c.282C>T, p.(Cys94*)	Ex 6	p.(Cys94*)	PD	T-118.03/*de novo*	Very strong evidence for pathogenic (PVS1)
Previously reported PubMed: 16712695	c.300G>A	Ex 6	p. (Trp100*)	PD	T-184.03/*de novo*	Very strong evidence for pathogenic (PVS1)
Previously reported PubMed: 7951315	c.307C>T	Ex 6	p.(R103*)	PD	AN210/*de novo*	Very strong evidence for pathogenic (PVS1)

novel	c.313del	Ex 6	p.(Arg105Aspfs*19)	PD	AN211/*de novo*	Very strong evidence for pathogenic (PVS1)
Previously reported PubMed: 8364574	c.357+1G>A	Int 7	r.spl	–	T-178.01/fam(pat), T-187.03/fam(pat)	Very strong evidence for pathogenic (PVS1)
novel	c.357+1_357+5del/insA	Int 7	r.spl	–	AN197/*de novo*	Very strong evidence for pathogenic (PVS1)
novel	c.486delinsTC	Ex 7	p.(Trp162Cysfs*38)	LNK	T-164.03/*de novo*	Very strong evidence for pathogenic (PVS1)
novel	c.626dup	Ex 8	p.(Leu210Alafs*5)	–	T-119.03/fam(mat)	Very strong evidence for pathogenic (PVS1)
novel	c.597_619dup	Ex 8	p.(Lys207Argfs*8)	LNK	T-174.03/fam(mat)	Very strong evidence for pathogenic (PVS1)

Previously reported PubMed: 7550230	c.607C>T	Ex 8	p.(Arg203*)	LNK	AN180/de novo , T-146.03/de novo , T-175.03/de novo , T-171.03/*de novo*	Very strong evidence for pathogenic (PVS1)
Previously reported PubMed: 32360764	c.681A>G	Int 8	p.(Lys227=) r.(spl?)	HD	AN214/*de novo*	Likely pathogenic (PM2, PS2, PP5, PP4)
Previously reported PubMed: 34101622	c.683-6T>A	Int 8	r.(spl?)	–	T-152.03/*de novo*	Likely pathogenic (PM2, PS2, PP5, PP4)
novel	c.697delC	Ex 9	p.(His233Ilefs*11)	HD	AN177/*de novo*	Very strong evidence for pathogenic (PVS1)
Previously reported PubMed: 1345175	c.718C>T	Ex 9	p.(Arg240*)	HD	AN189/*de novo* , T-190.03/fam(mat)	Very strong evidence for pathogenic (PVS1)
novel	c.765G>A	Ex 9	p.(Gln255=) r.(spl?)	HD	A-6/*de novo*	Likely pathogenic (PM2, PS2, PP5, PP4)

Previously reported PubMed: 10234503	c.781C>T	Ex 10	p.(Arg261*)	HD	T-149.03/*de novo*	Very strong evidence for pathogenic (PVS1)
novel	c.805G>T	Ex 10	p.(Glu269*)	HD	T-186.03/*de novo*	Very strong evidence for pathogenic (PVS1)
Previously reported PubMed: 25525159	c.916+1G>C	Int 10	r.spl	–	97.03/*de novo*	Very strong evidence for pathogenic (PVS1)
novel	c.919del	Ex 11	p.(Ser307Profs*58)	PST	T-206.03/fam(mat)	Very strong evidence for pathogenic (PVS1)
Previously reported PubMed: 8111379	c.949C>T	Ex 11	p.(Arg317*)	PST	AN179/fam(mat), T-161.03/fam(mat), 82.03/*de novo*	Very strong evidence for pathogenic (PVS1)
Previously reported PubMed: 26661695	c.1033-2A>G	Int 11	r.spl	–	AN187/*de novo*	Very strong evidence for pathogenic (PVS1)

novel	c.1184-2_1184 delAGGinsGGA	Int 12	r.spl	–	AN147/*de novo*	Very strong evidence for pathogenic (PVS1)
Previously reported PubMed: 11309364	c.1268A>T p.Ter423Leu (X423L)	Ex 13	p.(*423Leuext*14)	PST	84.03/*de novo*	Very strong evidence for pathogenic (PVS1)
