# Supplementary material for: Relative Frequencies of PAX6 Mutational Events in a Russian Cohort of Aniridia Patients in Comparison with the World’s Population and the Human Genome
Source: Int J Mol Sci. 2022 Jun 15;23(12):6690. doi: 10.3390/ijms23126690 (PMC9223373; doi:10.3390/ijms23126690)
Supplement: Supplementary file 1 [file ijms-23-06690-s001.zip › Table S2.pdf]

Table S2. Summary of 15 newly reported 11p13 deletions.

**Proband(s)**	**Size**	**Genomic coordinates of hemizygous region (hg18)**	**Genes in the deletion region (partial list)**
69.03/*de novo,* 81.03/*de novo*	~7 481 kb	27 636 398 − 35 117 389	*BDNF–FSHB–DCDC1−ELP4−PAX6−RCN1−WT1−HIPK3−LMO2–CD44*
74.03/*de novo*, T-134.03/*de novo*, T-185.03/*de novo*	~4 907 kb	30 210 128 − 35 117 389	*FSHB–DCDC1−ELP4−PAX6−RCN1−WT1−HIPK3−LMO2–CD44*
60.03/*de novo*	~3 627 kb	30 210 128 − 33 837 614	*FSHB–DCDC1−ELP4−PAX6−RCN1−WT1−HIPK3−LMO2*
T-148.03/pat	~145 kb	31 628 232 − 31 772 814	*ELP4−PAX6*
AN169/*de novo*, AN183/*de novo*	~235 kb	31 628 232 − 31 863 698	*ELP4−PAX6*
AN174/*de novo*, 87.03/*de novo*, T-147.03/*de novo*, AN219/mat, T-181.03/*de novo*	~350 kb	31 285 887 − 31 628 232	*DCDC1−ELP4*
AN202/*de novo*	~922 kb	31 285 887 − 32 208 363	*DCDC1−ELP4−PAX6−RCN1*
AN190/mat	~13 kb	31 768 079 − 31 780 904	*PAX6*
AN175/*de novo*	~1 kb	31 771 789 − 31 772 814	*PAX6*
T-168.03/*de novo*	~2 kb	31 778 912 − 31 780 904	*PAX6*
69.03/*de novo*, 81.03/*de novo*	~7 481 kb	27 636 398 − 35 117 389	*BDNF–FSHB–DCDC1−ELP4−PAX6−RCN1−WT1−HIPK3−LMO2–CD44*
74.03/*de novo*, T-134.03/*de novo*, T-185.03/*de novo*	~4 907 kb	30 210 128 − 35 117 389	*FSHB–DCDC1−ELP4−PAX6−RCN1−WT1−HIPK3−LMO2–CD44*
60.03/*de novo*	~3 627 kb	30 210 128 − 33 837 614	*FSHB–DCDC1−ELP4−PAX6−RCN1−WT1−HIPK3−LMO2*

PAX6 coordinates: 31 762 916–31 789 477 (hg18)
